# Quality of Life of People Living with HIV/AIDS under the New Epidemic Characteristics in China and the Associated Factors

**DOI:** 10.1371/journal.pone.0064562

**Published:** 2013-05-31

**Authors:** Wei Sun, Ming Wu, Peng Qu, Chunming Lu, Lie Wang

**Affiliations:** 1 Department of Environmental Health, School of Public Health, China Medical University, Shenyang, Liaoning, PR China; 2 Department of Social Medicine, School of Public Health, China Medical University, Shenyang, Liaoning, PR China; 3 Liaoning Provincial Center for Disease Control and Prevention, Shenyang, Liaoning, PR China; 4 Liaoning Women and Children’s Health, Shenyang, Liaoning, PR China; University of Toronto, Canada

## Abstract

**Background:**

Improvement of quality of life has been one of goals in health care for people living with HIV/AIDS (PLWHA). In China, the epidemic characteristics have changed and transmission is now most commonly sexual contact. However, the assessment of quality of life of PLWHA under new characteristics has limited reporting. This study was designed to assess the quality of life among PLWHA who contracted disease mainly via sexual contact and to clarify the associated factors.

**Methods:**

A cross-sectional study was performed in Liaoning Province. Sample size (800) was calculated based on the fatality rate and enlarged with consideration on the loss of response. Participants were sampled by tables of random numbers among all registered PLWHA. Questionnaires pertaining to quality of life (SF-36) and related factors (demographic characteristics, social support and network, HIV/AIDS awareness, and behavior factors) were distributed during December 2010-April 2011. 783 effective responses were obtained.

**Results:**

The average scores of physical component summary (PCS), mental component summary (MCS), and total score (TS) were 66.8±21.9 (Mean±SD), 62.2±20.9, and 64.5±20.2. General linear model analysis revealed that, in standardized estimate (β) sequence, PCS was significantly associated with monthly income, perceived social support, antiretroviral therapy, transmission, and ethnicity; MCS was associated with perceived social support, antiretroviral therapy, condom use, monthly income, transmission, ethnicity, and alcohol consumption; whereas TS was associated with perceived social support, antiretroviral therapy, monthly income, transmission, condom use, and ethnicity.

**Conclusions:**

Quality of life for PLWHA who contracted HIV mainly via sexual contact was worse and both physical conditions and social integration were impacted. Under current epidemic characteristics, efforts to increase social support and enhance the implementation of supporting policy are necessary to improve the quality of life of PLWHA.

## Introduction

HIV/AIDS is a major health problem worldwide [1]. People living with HIV/AIDS (PLWHA) experience both deterioration of physical health and psychological stress resulting from the fear of AIDS, and the associated stigma, as well as from discrimination [2], [3]. Thus, the World Health Organization (WHO) and the Joint United Nations Programme on HIV/AIDS (UNAIDS) have proposed that improvement of quality of life should be one of the primary goals in providing care and support to PLWHA [4].

In China, the HIV/AIDS epidemic is growing, and its characteristics are changing. It is reported that there were 370,393 people infected by HIV/AIDS by October 2010 [5]. However, the National Center for AIDS/STD Control and Prevention, China CDC reported that perhaps even as many as half of that actual number of PLWHA may not yet be identified and/or included in statistics. Accordingly, estimations of the number of PLWHA in China have increased from 650,000 in 2005 to 780,000 in 2011, although the estimated prevalence of HIV remained rather low (0.050% in 2005, 0.054% in 2007, 0.057% in 2009 and 0.058% in 2011) [6]. In addition, since 2009, the most prominent route of transmission has changed to sexual contact [7], [8], and those people newly diagnosed with HIV/AIDS are concentrated in large and medium-sized cities [6]; whereas, prior to 2009, almost 100% of PLWHA were infected via contaminated blood products and resided in and around the rural area of Henan Province [7]. In fact, it has been reported that nearly 90% of PLWHA who contracted the disease via contaminated blood products were farmers and the percentage of those with more than 10 years of education was only 1.78% [9]. Although not heretofore well-documented, it is anticipated that the recent shift in epidemic characteristics has had a corresponding effect on the quality of life and associated factors for PLWHA. Thus, identifying the current risk factors that impact the quality of life among the current PLWHA population is necessary for providing proper health care and improving patients’ quality of life.

Studies related to quality of life for PLWHA have been conducted worldwide [10]–[17]. However, following the epidemic previous characteristics, studies performed in China have mainly focused on PLWHA who were infected by contaminated blood products [10]–[13]. Few have reported results conducted among PLWHA who contracted the disease mainly via sexual contact. According to our knowledge, the related study with the biggest sample size [15] was a survey performed among 289 HIV/AIDS patients in one hospital, which seemed to weaken the representation of the study population and limit the generalization of the study conclusions. Until now, a population-based study on the quality of life of PLWHA under the new epidemic characteristics has not yet been reported.

The present study was designed to assess the quality of life for PLWHA who contracted the disease mainly via sexual contact and to explore the associated factors. The population sample was selected from PLWHA in Liaoning Province, where the income level is similar to the national average according to the China Yearbook and the main transmission mode of HIV/AIDS is sexual contact as reported by Chinese CDC. Since the Short Form-36 (SF-36) has proved to be a reliable instrument for the assessment of quality of life of Chinese PLWHA [18], it was selected to indicate the quality of life in this study. As for the associated factors, previous studies have revealed sociodemographic characteristics (age, sex, ethnicity, sexual orientation, education, employment, and income), health behaviors (smoke and alcohol use), life stressors and coping abilities (antiretroviral therapy), and social support (e.g., neighborhood and family support) to be the primary risk factors affecting quality of life of PLWHA [12]–[17]. These factors were also examined in the present study. In addition, with consideration on the specialty of this disease, HIV/AIDS awareness and condom use were included in this study to clarify the associated factors to quality of life of PLWHA under the new epidemic characteristics.

## Methods

### Ethics Statement

The study protocol and informed consent form received ethics approval from the Committee on Human Experimentation of China Medical University and Liaoning Provincial Center for Disease Control and Prevention. Written informed consent concerning conduct of the survey was obtained from each participant. We protected the privacy of individuals in processing personal data and maintained confidentiality of individual records and accounts. The participation in this study did not affect the future free health examination and treatment which is standard in China.

### Study Area and Study Population

According to the Chinese Law on the Prevention and Treatment of Infectious Diseases, any unit must report to the Center for Disease Control and Prevention (CDC) once it finds an infectious disease, and any unit or individual must respond to the inquiries by health institutions and accept their related examinations and investigations. Once diagnosed, PLWHA will be registered with their provincial CDC, and all registered PLWHA must visit their respective provincial CDCs every three months for health examinations and to access free treatments, if necessary. Thus, the provincial CDC is the appropriate venue to conduct this study.

The present study was conducted in Liaoning Province, the commercial hub of northeast China with income levels similar to the national average, according to the China Yearbook. In Liaoning Province, transmission of HIV/AIDS occurs mainly via sexual contact, as reported by Chinese CDC. Participants were sampled by tables of random numbers among all PLWHA who were registered in Liaoning Provincial CDC by November 30, 2010 and met the inclusion criteria: (1) positive for HIV antibody, and (2) ranging in age from 18–65 years. The sample size (755) was calculated based on fatality rate (11.7% in 2009) according to the formula N = 100*(1-P)/P. It was increased to 800 in consideration of an anticipated loss of response. If an individual refused to participate, a replacement would be selected via the same process. While conducting this study, we recruited a total of 24 replacements. Questionnaires pertaining to SF-36 and related factors were distributed to all sampled individuals between December 2010 and April 2011 while they visited the CDC. To ensure the success of this investigation, the CDC officers who are in charge of HIV/AIDS epidemic were trained for two weeks to assist participants having difficulty understanding the questionnaire, then that was self-administered with assistance if required. A total of 783 effective responses (effective response rate 97.9%) were obtained.

### Measurements of Quality of Life

The SF-36 has proved to be a reliable assessment of quality of life of PLWHA with the Cronbach’s alpha ranging from 0.75 to 0.90 for the eight dimensions [18]. Thus, the SF-36 was used as the indicator of quality of life in this study. This form contained 36 items and measured eight different dimensions of health: physical functioning (PF), role limitation due to physical problems (RP), bodily pain (BP), general health perceptions (GH), vitality (VT), social functioning (SF), role limitation due to emotional problems (RE), and mental health (MH). These dimensions were further categorized into physical component summary (PCS) and mental component summary (MCS). PCS was drawn from PF, RP, BP and GH, whereas MCS was drawn from VT, SF, RE and MH. The Cronbach’s alpha for PCS and MCS in this study were 0.898 and 0.877, respectively. The total score (TS) of SF-36 that included both physical and mental health status was also calculated. The Cronbach’s alpha for TS in this study was 0.931. As for the validity, construct validity analysis showed that values of χ^2^/df, GFI, CFI, TLI and RMSEA were 4.93, 0.89, 0.93, 0.92 and 0.07 for PCS, 4.99, 0.94, 0.96, 0.94 and 0.07 for MCS, and 3.81, 0.86, 0.91, 0.90 and 0.06 for TS, which proved that PCS, MCS and TS used in this study were enough validated. The scores of PCS, MCS and TS ranged from 0 to 100.

### Measurements of Demographic Characteristics, Social Support and Network, HIV/AIDS Awareness and Behavioral Factors

*Demographic characteristics* included age, sex, ethnicity, marital status, education, job, monthly income and sexual orientation. The ethnicity was categorized as Han and others. As for marital status, only 4 people (0.5%) were widowed, and therefore this group was combined with the divorced group. Education was categorized as primary/middle school, high school, and junior college and over. Monthly income (yuan) was divided into < = 1000, 1001–2000, and >2000 groups. Sexual orientation was categorized as homosexuality, heterosexuality, bisexuality, and unknown groups.

*Social support and network* comprised 4 items: 1) living arrangement, 2) neighborhood, 3) family awareness of sexual orientation, and 4) perceived social support. Living arrangement was divided into “living with others” group and “living alone” group. Neighborhood was assessed by asking the question “How do you feel about your relationship with your neighbors?” with four possible responses: very good, good, fair and poor. The poor group was combined with fair group because of the low response rate (2.1%). Family awareness of sexual orientation was categorized according to the potential answers: yes or no/unsure. Perceived social support was measured using the Duke-UNC Functional Social Support Questionnaire (FSSQ), which is designed to measure an individual’s perception of social support network [19]. It included eight items and yielded a single total score. The Cronbach’s alpha coefficient in the present study was 0.920.

*HIV/AIDS awareness* included 4 items: 1) comprehensive HIV/AIDS knowledge, 2) transmission, 3) disease progression, and 4) antiretroviral therapy. Comprehensive HIV/AIDS knowledge was examined using the United Nations General Assembly Special Session (UNGASS) indicator [20] and could be defined as “yes” only if answers to all five questions are correct. Transmission was examined by asking the question “Do you know how you were infected by HIV?”. The responses were classified into drug injection, contaminated blood products, heterosexual contact, lesbian/gay/bisexual/trans/queer (LGBTQ) contact, and refuse/unknown groups. Disease progression was assessed according to the Diagnostic Criteria for HIV/AIDS [21]. People were defined as HIV infection only when HIV seropositivity was present and participants were defined as AIDS status when the CD4+ T lymphocyte count was less than 200×10^6^/L or at least one symptomatic infection had occurred in addition to HIV seropositivity. Antiretroviral therapy was assessed on the basis of the experience of receiving any type of antiviral medicine such as zidovudine, lamivudine, nevirapine, stavudine, etc. from the provincial CDC.

*Behavioral factors* referred 3 items: 1) smoking, 2) alcohol consumption, and 3) condom use at the last sexual behavior since HIV infection. Smoking was assessed by asking “Are you currently a smoker?” with three answers: yes, used to smoke but quit, and never. Alcohol consumption was measured by asking the question “Have you consumed alcohol in the last month?”. Condom use at the last sexual behavior since HIV infection examined the status of condom use after HIV diagnosis. The responses were divided into “yes” group, “have no sexual behavior” group, and “no” group.

### Statistical Analysis

Data analyses were performed separately for PCS, MCS and TS. In this study, there were 39 individuals (4.98%) who refused to answer or didn’t know the transmission route; thus, their data was excluded. As for missing data, because the missing ratios of all items were less than 5%, no adjustments were performed in this study. The distributions of PCS, MCS and TS in categorical variables were examined by the Student’s t-test and one-way ANOVA. Correlations of PCS, MCS and TS with continuous variables were examined by Pearson correlation. General linear model analysis was used to identify the factors associated with PCS, MCS and TS. All the variables with p<0.25 in the univariate analysis were entered into the model. With adjustment for age and sex, items with p>0.05 were eliminated one at a time in the sequence of p value. When an item was eliminated, if the change in any remaining parameter estimate was greater than 20%, the item would remain in the model as a confounder. In this study, no confounder was found during elimination. SAS for Windows, Ver. 8.2, was used for all statistical analyses.

In addition, the agreement among categorical variables was evaluated with the Kappa test. If Kappa value was more than 0.50, these variables were considered in agreement and would be adjusted in multivariate analysis. In this study, Kappa value between disease progression and antiretroviral therapy was 0.7671; therefore, these two variables were considered in agreement. A Pearson correlation was used to assess the co-line variables among continuous items. In this study, no co-line data were found.

## Results

### Characteristics of Study Population

By November 30, 2010, there were 2696 PLWHA registered at the Liaoning Provincial CDC who met our inclusion criteria (2358 men and 338 women). The average age of this special population was 35.2±10.9 years. The constituent ratios for the transmission mode were 6.0% for blood, 10.1% for injecting drug, 27.9% for heterosexual contact, 42.9% for LGBTQ contact, and 13.1% for others (refuse/unknown). In comparison with this target population, the average age of our sampled individuals was 37.5±11.3 years (701 men and 82 women) and the ratios infected by blood, injecting drug, heterosexual contact, LGBTQ contact and others were 1.8%, 7.0%, 28.1%, 58.1% and 5.0%, respectively. For further data analysis, the group who refused to answer or didn’t know their transmission route (39 PLWHA) was excluded. The participant characteristics are shown in Table 1.

**Table 1:** The participant characteristics (N = 744).

		N	%
**Demographic characteristics**			
Sex	Men	669	89.92
	Women	75	10.08
Ethnicity	Han	683	91.80
	Others	61	8.20
Marital status	Single	357	47.98
	Married/cohabitation	251	33.74
	Divorced/widow	136	18.28
Education	Primary/middle school	319	42.88
	High school	171	22.98
	Junior college and over	251	33.74
	Frequency missing	3	0.40
Job	Student/housework/unemployed	229	30.78
	Peasant/herder/fisherman	88	11.83
	Informal employees	172	23.12
	Formal employees	255	34.27
Monthly income (yuan)	< = 1000	327	43.95
	1001–2000	199	26.75
	>2000	206	27.69
Sexual orientation	Homosexuality	327	43.95
	Heterosexuality	213	28.63
	Bisexuality	97	13.04
	Unknown	93	12.50
	Frequency missing	14	1.88
**Social support and network**			
Living arrangement	Living with others	499	67.07
	Alone	241	32.39
	Frequency missing	4	0.54
Neighborhood	Very good	295	39.65
	Good	173	23.25
	Fair/poor	269	36.16
	Frequency missing	7	0.94
Family awareness of sexual orientation	Yes	261	35.08
	No/unsure	446	59.95
	Frequency missing	37	4.97
**HIV/AIDS awareness**			
Comprehensive HIV/AIDS knowledge	Yes	455	61.16
	No	289	38.84
Transmission	Injecting drug	55	7.39
	Blood	14	1.88
	Heterosexual	220	29.57
	LGBTQ	455	61.16
Disease progression	HIV infection	508	68.28
	AIDS	236	31.72
Antiretroviral therapy	Yes	255	34.27
	No	480	64.52
	Frequency missing	9	1.21
**Behavioral factors**			
Smoking	Never	378	50.81
	Quit	59	7.93
	Yes	299	40.19
	Frequency missing	8	1.08
Alcohol consumption	No	419	56.32
	Yes	313	42.07
	Frequency missing	12	1.61
Condom use at the last sexual behavior since HIV infection	Yes	529	71.10
	Have no sexual behavior	133	17.88
	No	64	8.60
	Frequency missing	18	2.42
		**N**	**Mean±SD**
**Demographic characteristics**			
Age		744	37.1±11.1
**Social support and network**			
Perceived social support		744	3.0±1.1

### The Assessment of Quality of Life

Scores of eight dimensions of SF-36 among PLWHA and the comparisons with other Chinese populations are shown in Table 2. Among our participants, the highest score was PF (Mean±SD: 83.0±21.5) and the lowest score was GH (Mean±SD: 45.2±21.0). The comparisons with the standards Chinese general men and the AIDS patients who were infected by contaminated blood products were performed.

**Table 2:** Scores of eight dimensions of SF-36 among PLWHA and the comparisons with other Chinese populations (N = 744).

	This study	Male standard [^23^]	Male AIDS patients receiving HAART [^24^]
	Mean±SD	Mean±SD	Mean±SD
Physical functioning	83.0±21.5	84.4±18.6	86.2±18.8*
Role limitation due to physical problems	62.8±42.7	82.4±32.6**	51.3±44.7**
Bodily pain	76.2±25.1	83.0±19.0**	71.8±25.8*
General health perceptions	45.2±21.0	58.0±19.9**	43.3±21.0
Vitality	53.7±21.0	53.8±20.9	54.9±20.3
Social functioning	75.6±19.2	83.1±17.5**	56.5±16.7**
Role limitation due to emotional problems	61.5±43.9	84.3±32.3**	48.2±44.6**
Mental health	58.3±19.2	60.3±23.0	59.3±19.5

* p<0.05;

** p<0.01.

### Univariate Analysis

The univariate analysis of factors related to quality of life is shown in Table 3. The average scores of PCS, MCS and TS of the sampled PLWHA were 66.8±21.9 (Mean±SD), 62.2±20.9 and 64.5±20.2, respectively. The univariate analysis showed that, among categorical variables, Han ethnicity, single, higher education, having a job, higher monthly income, nice neighborhood, transmission by sexual contact, HIV infection status, condom use at the last sexual behavior since HIV infection, and taking antiretroviral therapy groups have significantly higher levels of PCS, MCS and TS. In addition, PCS was also correlated with sexual orientation (known), comprehensive HIV/AIDS knowledge (yes) and smoking (never); MCS was also significantly correlated with alcohol consumption (yes); whereas TS was related to sexual orientation (known), comprehensive HIV/AIDS knowledge (yes), smoking (never), and alcohol consumption (yes). Pearson correlation analysis indicated that all PCS, MCS, and TS have significantly negative correlations with age and positive correlations with perceived social support.

**Table 3:** The univariate analysis of factors related to quality of life.

		PCS	MCS	TS
		Mean±SD	Mean±SD	Mean±SD
		66.8±21.9	62.2±20.9	64.5±20.2
**Demographic characteristics**				
Sex	Men	67.2±21.9	62.7±21.0	64.9±20.3
	Women	63.5±21.6	58.3±19.6	60.9±19.2
Ethnicity	Han	67.4±21.8*	62.9±20.9**	65.1±20.1**
	Others	60.7±22.8	54.8±19.8	57.8±20.1
Marital status	Single	69.7±19.8**	64.6±20.1**	67.2±18.6**
	Married/cohabitation	66.8±23.1	62.6±20.9	64.7±20.9
	Divorced/widow	59.1±23.1	55.5±21.7	57.3±21.2
Education	Primary/middle school	63.2±23.1	60.2±21.3	61.7±21.0
	High school	67.4±21.5	62.6±20.2	65.0±19.7
	Junior college and over	71.3±19.7**	64.9±20.5*	68.1±18.9**
Job	Student/housework/unemployed	59.4±23.9	57.3±21.5	58.4±21.5
	Peasant/herder/fisherman	71.6±20.3	65.1±22.1	68.4±19.8
	Informal employees	71.7±19.7**	65.7±19.1**	68.7±18.3**
	Formal employees	68.5±20.3	63.4±20.3	66.0±19.1
Monthly income (yuan)	< = 1000	59.9±22.8	58.2±21.4	59.1±21.0
	1001–2000	69.7±19.6	63.5±19.8	66.6±18.3
	>2000	74.9±18.8**	67.1±19.8**	71.0±18.2**
Sexual orientation	Homosexuality	69.7±20.3	63.8±20.4	66.7±19.2
	Heterosexuality	64.9±22.9	60.8±21.0	62.8±20.9
	Bisexuality	67.2±20.2	62.2±21.9	64.7±19.7
	Unknown	61.4±24.4**	60.4±21.1	60.9±21.5*
**Social support and network**				
Living arrangement	Living with others	66.1±22.2	61.6±21.3	63.8±20.6
	Alone	68.3±21.1	63.6±20.0	65.9±19.4
Neighborhood	Very good	70.4±20.8**	65.9±21.1**	68.1±19.8**
	Good	67.1±20.6	62.1±18.9	64.6±18.6
	Fair/poor	62.5±23.3	58.4±21.3	60.5±21.1
Family awareness of sexual orientation	Yes	66.5±22.5	62.1±20.7	64.3±20.4
	No/unsure	67.5±21.3	62.5±21.0	65.0±20.0
**HIV/AIDS awareness**				
Comprehensive HIV/AIDS knowledge	Yes	68.2±21.7*	63.3±20.7	65.8±20.1*
	No	64.6±22.1	60.5±21.0	62.6±20.3
Transmission	Injecting drug	49.8±24.2	49.3±22.2	49.6±21.3
	Blood	58.5±16.0	62.2±15.3	60.3±13.5
	Heterosexual	67.1±22.0	62.2±20.8	64.7±20.4
	LGBTQ	69.0±20.8**	63.8±20.4**	66.4±19.4**
Disease progression	HIV infection	69.6±21.0**	63.8±20.2**	66.7±19.4**
	AIDS	60.9±22.7	58.9±21.8	59.9±21.1
Antiretroviral therapy	Yes	59.9±21.8	56.9±21.6	58.4±20.5
	No	70.6±21.0**	65.1±20.0**	67.9±19.3**
**Behavioral factors**				
Smoking	Never	69.0±20.9*	63.3±20.5	66.1±19.6*
	Quit	61.8±21.6	57.1±21.7	59.5±20.6
	Yes	65.0±23.1	61.8±21.2	63.4±20.8
Alcohol consumption	No	65.8±22.9	60.0±22.4	62.9±21.4
	Yes	68.1±20.7	65.0±18.5**	66.6±18.5*
Condom use at the last sexual behavior since HIV infection	Yes	68.9±21.2**	64.4±20.3**	66.7±19.6**
	Have no sexual behavior	60.9±24.3	57.4±23.1	59.2±22.2
	No	61.7±20.9	55.4±19.4	58.6±19.1
		**Pearson ** ***r***	**Pearson ** ***r***	**Pearson ** ***r***
**Demographic characteristics**				
Age		−0.16**	−0.12**	−0.15**
**Social support and network**				
Perceived social support		0.28**	0.32**	0.32**

* p<0.05;

** p<0.01.

### Multivariate Analysis

The general linear model analysis for identifying the factors associated with quality of life is shown in Table 4. The items entered the model included age, sex, ethnicity, education, job, monthly income, sexual orientation, living arrangement, neighborhood, comprehensive HIV/AIDS knowledge, transmission, disease progression (or antiretroviral therapy), smoking, alcohol consumption, condom use at the last sexual behavior since HIV infection, and perceived social support. With adjustment for age and sex, PCS were significantly associated with, in standardized estimate (β) sequence, monthly income, perceived social support, antiretroviral therapy, transmission, and ethnicity; MCS was significantly associated with perceived social support, antiretroviral therapy, condom use in the last sexual behavior since HIV infection, monthly income, transmission, ethnicity and alcohol consumption; and TS was significantly associated with perceived social support, antiretroviral therapy, monthly income, transmission, condom use at the last sexual behavior since HIV infection, and ethnicity. The model R^2^ for PCS, MCS and TS were 0.2417, 0.2188 and 0.2491, respectively.

**Table 4:** The general linear model analysis for identifying the factors associated with quality of life.

		Parameterestimate	Standardizedestimate (β)	*p* value
**PCS (N = 714):**				
Intercept		60.6185		
Age^a^		−0.0987	−0.0499	0.1616
Sex^a^	(Men vs Women)	0.6944	0.0095	0.7965
Monthly income	(< = 1000 vs >2000)	−11.745	−0.2659	<.0001
	(1001–2000 vs >2000)	−5.3396	−0.1084	0.0063
Perceived social support		4.6396	0.2238	<.0001
Antiretroviral therapy	(Yes vs No)	−10.3254	−0.2233	<.0001
Transmission	(Blood vs LGBTQ)	−7.892	−0.0462	0.1705
	(Heterosexual vs LGBTQ)	1.0186	0.0211	0.5809
	(Injecting drug vs LGBTQ)	−14.9394	0.1814	<.0001
Ethnicity	(Han vs Others)	7.1077	0.0884	0.0079
**MCS (N = 695):**				
Intercept		39.6655		
Age^a^		−0.0073	−0.0038	0.9182
Sex^a^	(Men vs Women)	1.8577	0.0269	0.4854
Perceived social support		5.742	0.2888	<.0001
Antiretroviral therapy	(Yes vs No)	−8.9567	−0.2012	<.0001
Condom use at the last sexual behavior since HIV infection	(Yes vs No )	7.1668	0.1517	0.0055
	(Have no sexual behavior vs No)	3.6148	−0.0667	0.2209
Monthly income	(< = 1000 vs >2000)	−5.5959	−0.132	0.0015
	(1001–2000 vs >2000)	−3.871	−0.0816	0.0452
Transmission	(Blood vs LGBTQ)	0.7798	0.004	0.8941
	(Heterosexual vs LGBTQ)	0.9667	0.0209	0.5975
	(Injecting drug vs LGBTQ)	−9.5497	−0.1214	0.001
Ethnicity	(Han vs Others)	7.8498	0.1031	0.0028
Alcohol consumption	(No vs Yes)	−3.8167	−0.0896	0.0096
**TS (N = 701):**				
Intercept		46.8024		
Age^a^		−0.0361	−0.0195	0.5904
Sex^a^	(Men vs Women)	1.6861	0.0252	0.4996
Perceived social support		5.164	0.2688	<.0001
Antiretroviral therapy	(Yes vs No)	−9.8436	−0.2295	<.0001
Monthly income	(< = 1000 vs >2000)	−8.7474	−0.2138	<.0001
	(1001–2000 vs >2000)	−4.4594	−0.0975	0.0144
Transmission	(Blood vs LGBTQ)	−3.0597	−0.0195	0.5651
	(Heterosexual vs LGBTQ)	0.9648	0.0216	0.5756
	(Injecting drug vs LGBTQ)	−11.597	−0.1521	<.0001
Condom use at the last sexual behavior since HIV infection	(Yes vs No )	5.6732	0.1244	0.0198
	(Have no sexual behavior vs No)	2.0595	0.0395	0.458
Ethnicity	(Han vs Others)	7.2693	0.0985	0.0034

a: age and sex were fixed in the model.

The model R^2^ was 0.2417 for PCS, 0.2188 for MCS and 0.2491 for TS.

## Discussion

To our knowledge, this study was the first attempt to perform a population-based assessment of the quality of life among PLWHA under the new epidemic characteristics. The sample size was calculated and enlarged and all participants were randomly sampled from all PLWHA registered in Liaoning Province. Thus, we believe our study population is a good representation of PLWHA in Liaoning Province. Specially, 86.2% of our participants contracted HIV/AIDS via sexual contact and 58.1% of those cases were attributed to LGBTQ contact, coinciding with the current prominent transmission mode and the fact that men having sex with men accounted for 32.5% of all new HIV cases reported in 2009 [8],[22]. Also, the average age of our study population was 37.1 years, similar to the national average age of 38 years [7]. All these facts increase the generalization of our conclusions.

In comparison with the PLWHA who contracted the disease via contaminated blood products, the characteristics of our PLWHA population were quite different. Among previous PLWHA, nearly 90.1% were farmers and only 1.78% had received education of more than 10 years [9]; whereas 34.3% of our participants had formal occupations and 33.7% had graduated from junior college or over (≥15 years). Acknowledging these significant differences, it is necessary to assess the quality of life among PLWHA under the new epidemic characteristics and to clarify the current risk factors involved in order to provide health care properly.

To assess the quality of life of PLWHA under new epidemic characteristics, we compared our results with the studies conducted among Chinese general population (1688 participants and 51.9% aged 18–44 years) [23] and among AIDS patients who contracted via contaminated blood products and were receiving HAART (1194 participants and 53.0% aged 31–40 years) [24]. Since 90% of our participants were men, we mainly compared our results with the previous findings drawn from male participants. We found that levels of physical functioning, vitality, and mental health in our study population were same as that in the Chinese general population, which might be due to the fact that only one third of our participants (31.7%) were in the AIDS stage. Moreover, because all AIDS patients may receive free antiretroviral therapy in China [25], significant declines in physical functioning, vitality, and mental health among those being treated might not have ensued. However, the role limitation due to physical problems, bodily pain, general health perceptions, social functioning, and the role limitation due to emotional problems were considerably lower for our study population than for men in general population, but much higher than for AIDS patients who contracted HIV via contaminated blood products and were receiving HAART, even though the average age of the PLWHA in our study was higher than the ages of the other two study populations. These results revealed that, even if the health status of PLWHA who contracted the disease via sexual contact has not yet seriously deteriorated, their abilities in both physical condition and social integration were already affected. This phenomenon calls for particular attention when providing health care for PLWHA under the new epidemic characteristics.

With respect to the risk factors, it is well known that PLWHA suffer from serious stigmas and discrimination [2], [3]. Accordingly, any support tends to have a substantial beneficial effect on PLWHA. Regardless of coincidence of indicators of quality of life and social support used, this conclusion had been reported in both Chinese and foreign studies [12], [15], [26], [27]. In the present study, social support was found to have the strongest effect on MCS and TS, while for PCS, social support was the second strongest risk factor. These findings indicated that social support could be the most important risk factor for the quality of life of PLWHA who contracted HIV/AIDS via sexual contact. Because the majority of PLWHA who contracted the disease via sexual contact were the men who had sex with men [8], [22] and homosexuality has not yet been accepted in China, the discrimination towards these PLWHA was expected to be more serious than that towards commercial blood donors. As a result, the PLWHA who contracted the disease via sexual contact seemed to be more prone to isolation and loneliness, which inevitably worsens their quality of life. In addition, we measured perceived social support, rather than objective indictors, to study the effect of social support on quality of life. The precision of assessing social support indicated by a recipient’s perception of its strength could be exaggerated in comparison to any objective indicator. Thus, the strongest effect of social support on the quality of life of PLWHA could be observed in our study. The more extensive social support was perceived to be, the better the quality of life the PLWHA experienced. In view of the fact that PLWHA tended to conceal their health conditions and avoid social integration, efforts aimed at increasing social support, particularly lessening the fear of AIDS, changing attitudes towards PLWHA, and providing empathetic health and social care to PLWHA, might have considerable effect on the improvement of quality of life of this special population.

Antiretroviral therapy was found to be the second strongest risk factor to quality of life of PLWHA in this study. With consideration of other risk factors, the PLWHA who had taken antiretroviral therapy had lower levels of PCS, MCS and TS than PLWHA who had not yet begun to take such therapy, coinciding with the conclusion reported by Yan [14]. In China, all PLWHA are covered by “Four Frees and One Care” policy that has been implemented since March 2006 [25], and those with lower CD4+ T lymphocyte levels are able to receive free antiretroviral therapy at the provincial CDC. However, it also revealed that all PLWHA receiving antiretroviral therapy were in an advanced stage of the disease. We examined the agreement between antiretroviral therapy and disease progression and found that the Kappa value was 0.7671, much higher than 0.5. However, the model R^2^ was higher when antiretroviral therapy entered the model than the value when disease progression entered the model (0.2224 for PCS, 0.1944 for MCS and 0.2234 for TS). Thus, we determined the antiretroviral therapy to be as a risk factor for PCS, MCS and TS. Because this intervention is provided only when a PLWHA’s CD4+ T lymphocyte level reaches a certain low level, there is potential for clinical intervention to explore the suitable performance standard of antiretroviral therapy, i.e., using the antiretroviral medication and other supports to maintain physical and mental health conditions as early as possible would most likely make a significant contribution to sustaining a higher quality of life among PLWHA.

Demographic characteristics such as age, sex, education, income, marital status, ethnicity, etc. have been reported to be associated with quality of life of PLWHA [13]–[15], [24]. In the present study, monthly income and ethnicity, with adjustment for age and sex, were also found to be associated with PCS, MCS, and TS. Interestingly, monthly income was found to be the strongest demographic risk factor to PCS. Economic status is the base of nutrition and social activities. For the participants in this study, the lower their monthly income was, especially those who earned lower than 1000 yuan, the worse both their physical health and mental health became. The same conclusion was also obtained in another study conducted among 155 PLWHA who contracted disease mainly via sexual contact [14]. As for ethnicity, the majority group in China is Han. Both our study and the study conducted among blood donor in China [13] found that those with Han ethnicity had higher levels of quality of life than other ethnic groups. The reason for this ethnicity-related difference was not well understood. Perhaps it is due to the specificity of lifestyles, eating habits, and religious beliefs of minority ethnicities, which might reflect their social vulnerability and restrict their access to the healthcare, as found among minority students [28]. More detailed study is needed.

As for transmission, although the majority of our participants were PLWHA infected by sexual contact, 7.4% of participants were transmitted HIV/AIDS via injecting drugs. These PLWHA had the lowest levels of PCS, MCS, and TS scores than others in both univariate and multivariate analyses. Because drugs profoundly affect both the physical and mental well-being of the users, these results are not surprising. Moreover, because drug users have been one of special populations with a high risk of HIV infection due to intravenous drug use, needle sharing, and high-risk sexual behavior [29], these PLWHA are potentially important to target for care and support, not only for their quality of life but also for control of the HIV/AIDS epidemic. With respect to the transmission of HIV via contaminated blood products, a significantly different effect on quality of life between this mode and the current primary transmission mode, i.e., sexual contact, was not observed in our study. It may due to the fact that so few of our study population (1.9%) reported transmission via blood products, which may have weakened the comparison between those modes.

In addition, behavioral factors including alcohol consumption and condom use at the last sexual behavior since HIV infection were found to be able to affect quality of life of PLWHA. Alcohol consumption seems to act as a consolation for people with HIV/AIDS to release their mental stress, thus its effect on MCS was observed. As for condom use, it was found to have significant association with both MCS and TS. Condom use is an important preventive method for the spread of HIV/AIDS, especially now that sexual contact has become the most prominent mode of HIV transmission [7], [8]. However, many PLWHA considered condom use unnecessary for them due to their already-infected status [30]. The recipients with this opinion were also prone to hopelessness and the mental problems. Thus, PLWHA who did not use a condom at sexual behavior had lower levels of quality of life, especially mental health, than those who did use a condom. Furthermore, we found that nearly one tenth of PLWHA in Liaoning Province, where sexual contact has become the dominant transmission mode, did not use condoms. It constitutes a threat to the prevention and control of the HIV/AIDS epidemic. Therefore, targeting condom use to seek a compliance rate of 100% among PLWHA should be an urgent issue not only for their quality of life but for the interventions against the spread of HIV/AIDS epidemic.

There were several limitations for the present study. First, as the first attempt, this study included measurements of some risk factors, such as smoking, alcohol consumption and antiretroviral therapy, that were simplistic and broad, which might weaken the assessments of their effects. Second, all the individuals were sampled from the PLWHA registered in Liaoning Provincial CDC. However, according to the estimation reported by the National Center for AIDS/STD Control and Prevention, China CDC, nearly half of the HIV-positive people in Liaoning Province might not yet be identified. It might be the reason why heterosexual transmission accounted for 28.1% of the study population whereas the female PLWHA was paucity. The Chinese government has struggled to identify and register as many PLWHA as possible. Third, as the first attempt, we mainly focused on the random while sampling and the geographical distribution was not considered, which might limit the ability to generalize the conclusions. Fourth, although the present study was the first population-based study on the quality of life among PLWHA under the new HIV/AIDS epidemic characteristics, it was limited by its cross-sectional design. We are unable to draw any causal conclusion between quality of life and the risk factors. All findings obtained in this study need to be confirmed in a future prospective study. Besides, the intervention studies focusing on social support, access to healthcare, clinical preventive treatments, and improvement of healthcare workers along with the change of the primary transmission mode could be critical for the control of HIV/AIDS epidemic in light of our findings.

In conclusion, this study is the first attempt to perform a population-based survey to assess quality of life among PLWHA under the new characteristics of the HIV/AIDS epidemic and to clarify the associated factors. Our results indicated that quality of life of PLWHA who contracted the disease mainly via sexual contract was worse than quality of life in the Chinese general population. Social support had a considerable effect on their quality of life. Antiretroviral therapy, economic condition, transmission mode, and condom use were also associated with quality of life. Alcohol consumption affected the mental health status of this special population. All these findings suggested that increasing social support should be a priority while providing health care to PLWHA to achieve the improvement of their quality of life. Efforts on enhancing “Four Frees and One Care” policy and condom intervention would also contribute to the improvement of quality of life and the prevention and control of HIV/AIDS epidemic.
